# Topical Ophthalmic Formulation of Trichostatin A and SurR9-C84A for Quick Recovery Post-alkali Burn of Corneal Haze

**DOI:** 10.3389/fphar.2017.00223

**Published:** 2017-05-05

**Authors:** Kislay Roy, Prasad Neerati, Chun Hei Antonio Cheung, Rupinder K. Kanwar, Rajat Sandhir, Jagat R. Kanwar

**Affiliations:** ^1^Nanomedicine-Laboratory of Immunology and Molecular Biomedical Research, Centre for Molecular and Medical Research, School of Medicine, Faculty of Health, Deakin University, Geelong VIC, Australia; ^2^Drug Metabolism and Clinical Pharmacokinetics Division, Department of Pharmacology, University College of Pharmaceutical Sciences, Kakatiya University, Warangal, India; ^3^Department of Pharmacology and Institute of Basic Medical Sciences, College of Medicine, National Cheng Kung University, Tainan, Taiwan; ^4^Department of Biochemistry, Panjab University, Chandigarh, India

**Keywords:** survivin, haze, scarring, wound healing, cytokines, trichostatin-A

## Abstract

Alkali burn injury is a true ocular emergency of the conjunctiva and cornea that requires immediate precision. Lack of an immediate therapy can lead to a substantial damage in the ocular surface and anterior segment further causing visual impairment and disfigurement. We explored the regenerative capability of dominant negative survivin protein (SurR9-C84A) and histone deacetylase inhibitor trichostatin-A (TSA) *in vivo*, in a rat alkali burn model. A topical insult in rat eyes with NaOH led to degradation of the conjunctival and corneal epithelium. The integrity of the conjunctival and corneal tissue was increased by TSA and SurR9-C84A by improving the clathrin and claudin expressions. Wound healing was initiated by an increase in TGF-beta-1 and, increased endogenous survivin which inhibited apoptosis post-TSA and SurR9-C84A treatments. Protein expressions of fibronectin and alpha-integrin 5 were found to increase promoting corneal integrity. The cytokine analysis confirmed increased expressions of IL-1beta, IL-6, IL-12, IL-13, IFN-gamma, TNF-alpha, GMCSF, Rantes, and MMP-2 in injured cornea, which were found to be significantly downregulated by the combined treatment of SurR9-C84A and TSA. The ocular and systemic pharmacokinetic (PK) parameters were measured post-topical ocular administration of TSA and SurR9-C84A. The SurR9-C84A and TSA sustained relatively longer in the cornea, conjunctiva, and aqueous humor than in the tear fluid and plasma. Our results confirmed that a combination of TSA with SurR9-C8A worked in synergy and showed a promising healing and anti-inflammatory effect in a very short time against alkali burn. Therefore, a combination of TSA and SurR9-C84A can fulfill the need for an immediate response to wound healing in alkali burnt cornea. We also synthesized ultra-small chitosan nanoparticles (USC-NPs) targeted with alpha-SMA antibodies that can be used for delivery of TSA and SurR9-C84A specifically to the ocular burn site.

## Introduction

Alkali burns of eye account for 11.5–22.1% of all ocular traumas (Clare et al., 2012). Nearly two-thirds of such injuries occur in young men. Alkali burns are reported to occur more frequently than acid injuries (Wagoner, 1997). An alkali burn of the cornea often leads to an extensive damage resulting in a permanent visual impairment. The graveness of any ocular injury generally depends on factors such as toxicity of the chemical, exposure to chemicals, depth of penetration and the area of injury. As alkaline agents are generally lipophilic in nature, therefore, they can penetrate the tissues more rapidly than acids. Roper Hall and Dua classification system is the most widely accepted grading used to determine the level of degree of corneal, conjunctival and limbal acute ocular burns (Gupta et al., 2011). Alkali’s have the ability to saponify the fatty acids of cell, cell membranes in order to penetrate the corneal stroma, and destroy proteoglycans and collagen content of the cell. The damaged tissues further secretes proteolytic enzymes and inflammatory cytokines that lead to enhanced damage of the eye (Fish and Davidson, 2010). The current focus of medical strategies is to enhance the recovery of corneal epithelium, supplement collagen synthesis, minimizing collagen breakdown and controlling inflammation simultaneously (Asbell and Khatib, 2011). Many therapeutics have been used to prevent the cytokines and agents that jeopardize the integrity of the eye post a chemical injury. These therapeutics include corticosteroids, heparin, collagenase inhibitors, contact lenses, fibronectin, conjunctival flaps and corneal transplantation (Wagoner, 1997). However, due to the lack of a patient-friendly treatment with an immediate effect in preventing damage induced by alkali burns, treatment of the alkali-burned eye continues to be a major challenge to the ophthalmologist (Singh et al., 2013).

It has been reported that a 2-min topical treatment of histone deacetylase (HDAC) inhibitor trichostatin A (TSA) on rabbit corneas subjected to -9 D PRK substantially reduced the corneal haze (Sharma et al., 2009). Systemic administration of TSA in the ocular surface post-alkali-burn in a mouse model was found to reduce the inflammatory and fibrotic responses mainly, by inhibition of Smad signal in mesenchymal cells. The mitigation of inflammatory responses was also induced by a failure in activation and recruitment of macrophages (Kitano et al., 2010). However, a higher dosage of TSA (100–300 ng) can affect the chromatin structure and cause alterations in the DNA replication patterns (Kemp et al., 2005). We synthesized a dominant negative protein (SurR9-C84A) which replaced the depleted endogenous survivin protein in normal brain cells, protected them from T cell neurotoxicity, oxidative stress, and helped in their proliferation (Baratchi et al., 2010, 2011a,b). Previously, we analyzed the non-toxic nature of both these compounds and their combination in a bovine corneal opacity alkali burn model where we standardized the induction of alkali burn with NaOH. Our findings uncovered that SurR9-C84A and TSA led to a substantial improvement in the transmittance of light, improved the *trans*-epithelial electrical resistance (TEER) and reduced the ocular inflammation. Therefore, indicating the wound healing potential of this formulation for topical applications in cases of alkali burn (Bhasker et al., 2015; Lin et al., 2016).

Recently, we used these formulations in an alkaline burn rabbit models (Roy et al., 2016). Further to extend the published study we validated this work in another “rat model of alkali burn and haze/scarring.” In the present study, we want to use the rat model of scarring/haze to study, a number of biomarker reagents for rats are readily available than rabbits (pathologic markers, cytokine and apoptotic microarrays, primary and secondary antibodies) to study the complete mechanism which confirms/validate the pathways/molecules involved in humans. Therefore, in the present study, we have made an attempt to evaluate the anti-inflammatory and protective effects of SurR9-C84A, TSA and their combination in altering the alkali burn adverse events in a rat model. We also synthesized and characterized targeted nanoparticle system for targeted delivery of TSA and SurR9-C84A.

## Materials and Methods

### Reverse Transcription-Polymerase Chain Reaction to Evaluate the Effective Dose Range of Therapeutics

This procedure was carried out using a previously published protocol (Lin et al., 2016). Rat eyes were harvested from humanely killed rats and washed with HBSS. Rat eyes were further incubated with 1 M NaOH for 1 min and then treated with TSA (0.002, 0.02, and 0.1 g/kg), SurR9-C84A (0.5, 1.2, and 2.5 mg/kg), pSur/As-Sur (200, 200, and 800 μg/kg) and empty plasmid (200, 400, and 800 μg/kg) for 40 min. Untreated eyes were maintained as controls. After treatment period, eyes were washed with PBS and total RNA was extracted using the TRIzol^®^ reagent (cat# 15596-026, Thermo Fisher Scientific, Waltham, MA, USA), and complementary DNA (cDNA) was synthesized from RNA using the RevertAid H Minus First Strand cDNA Synthesis Kit (cat# K1631, Thermo Fisher Scientific). Expression of survivin, TGF-beta-1, alpha-SMA and collagen IV was determined by polymerase chain reaction (PCR).

### Rat Alkali Burn Model

This study was specifically approved by Deakin University Animal Ethics Committee, Geelong under the ethics application G12-2014. Seventy Wistar rats aged 10–12 weeks, were used for this study as this model is well established in the literature for corneal studies. The animals were housed and maintained in the upper animal house Facility at the Deakin University, Waurn Ponds campus, Geelong, VIC, Australia. Before starting the treatments and disease induction, all the animals were thoroughly examined for any signs of corneal abnormality. Before inducing the general anesthesia, the rats were placed in a cage with oxygen and pre-oxygenated.

**FIGURE 1:**
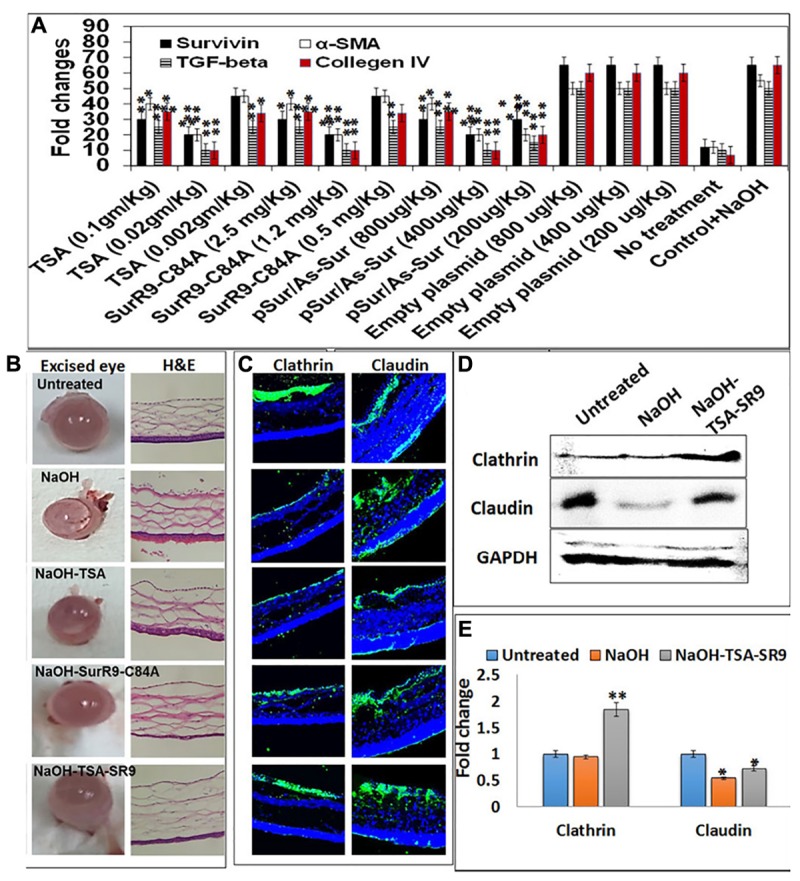
**Change in corneal tissue integrity post-alkali burn: (A)** Gene expression analysis to determine the effective dosage of TSA and SurR9-C84A using *ex vivo* rat eyes. **(B)** Rat eyes were exposed to 1 M NaOH for 1 min and then treated with TSA or SurR9-C84A or a combination of both [TSA+SurR9-C84A(SR9)] for 40 min. The excised eyes were used to determine the opacity and inflammatory scores and H&E staining was performed to determine the effect of alkali burn and treatments on corneal tissue integrity. **(C)** Immunofluorescence staining for expression of clathrin in conjunctival layer and claudin in corneal layer was performed to determine the effect of alkali burn and treatments on corneal tissue integrity. **(D)** Western blotting analysis confirmed that both decrease in expressions of clathrin and claudin in rat corneal tissue lysates. Each blot was repeated thrice and a representative blot has been presented. **(E)** Band density analysis of western blot was performed using image J software. The experiments were conducted in five animals per treatment group. The H&E staining was performed on five slides obtained from each animal and a representative image has been presented. Five images of each treatment were taken for immunofluorescence studies and a representative image has been presented. ^∗^*p* < 0.05, ^∗∗^*p* < 0.01, and ^∗∗∗^*p* < 0.001.

Rat anesthesia was induced using a combination of propofol (100 mg/kg), medetomidine (0.1 mg/kg), and fentanyl (0.1 mg/kg) injected intraperitoneally. Once anesthesia was induced the Isoflurane was administered at 1.5–2%. A 2.5 mm diameter filter paper, soaked in 1 M NaOH solution (pH 13.6), and was placed over the central cornea in the right eye of each animal. The excess/surplus fluid was discarded by a brief contact with a clean absorbent filter paper. The cornea was exposed for 1 min, the filter paper was discarded, and following which the ocular surface was rinsed with the isotonic Hanks balanced salt solution (HBSS). Cornea was then subjected to the topical application of TSA (0.02 g/kg-dissolved in PBS), SurR9-C84A (1.2 mg/kg-dissolved in PBS) and their combination (TSA+SurR9-C84A) for 40 min. This dosage was determined using a dose ranging study performed with rat eyes (**Figure 1A**). In all groups, only the right eye of each animal was treated. The left eye served as control and was treated with salt solution. Two milliliters blood was collected from the jugular venous catheter at (2, 10, and 30 min). Aqueous humor was collected post-humane killing of the animals. 100–150 μL sample of aqueous humor was withdrawn through a 27-gauge needle attached to a 1 ml syringe by puncture of the eyeball. All experiments were performed following the ethics approval G25-2014 and the guidelines from AECG.

### Histology Analysis

After the completion of the study, all tissues that required to be examined (eyes, brain, kidneys, liver, spleen, heart and lungs) were isolated. The cornea was carefully separated from the eye without damaging it and washed using phosphate buffer saline (PBS). Tissues were fixed using 4% paraformaldehyde (PF) overnight at 4°C and then washed in PBS. The tissue segments were dehydrated in graded ethanol, embedded in paraffin and 5–7 μm thick sections were cut using a microtome. Sections were collected on poly-lysine coated slides using the Leica cryostat and were fixed immediately in ice-cold acetone. All the sections were stained for haematoxylin–eosin (H&E) for critical analysis.

### Immunofluorescence Studies in Cells and Tissue Samples

Tissue slides were rehydrated by immersing in xylene (2 times for 10 min), 100, 95, and 70% ethanol for 5 min each, deionized water for 5 min. The antigen retrieval buffer (100 mM Tris, 5% w/v urea, pH 9.5) was pre-heated to 92–95°C. Slides were immersed in the pre-heated solution for 5–10 min. The tissues were encircled with a hydrophobic barrier using a barrier pen. Tissues were further blocked with 3% bovine serum albumin (BSA) for 1 h. The slides were washed and incubated with primary antibodies: mouse monoclonal anti-clathrin antibody (BD biosciences, 1:100 dilution), rabbit anti-claudin antibody (1:100 dilution, Santa Cruz, Australia), mouse monoclonal alpha-SMA antibody (Santa Cruz: 1:100 dilution), mouse monoclonal collagen type IV antibody (Santa Cruz: 1:100 dilution), D8-mouse monoclonal anti-survivin, (Santa Cruz: 1:100 dilution) for 1 h at 37°C. Post-washing thrice with PBS, the slides were incubated with fluorescein isothiocyanate (FITC) (anti-mouse, anti-rabbit FITC, Sigma–Aldrich) conjugated secondary antibody (1:100) for 1 h in dark. The slides were washed and mounting media with DAPI was added to the slides. The slides were viewed using Leica TCS SP5 laser scanning confocal microscope.

### Analysis of Pro and Anti-inflammatory Cytokines

The cytokine profiling was performed using the Rat Inflammatory Cytokines & Chemokines Multi-Analyte ELISArray Kit (QIAGEN, USA). Briefly, serial dilutions of the Antigen Standard and the experimental samples were prepared. 50 μL of Assay Buffer was added to each well of the 8-well ELISA strips. Fifty microliters samples and/or standards were added to the appropriate wells of the ELISA strips and incubated for 2 h at room temperature. The ELISA strips were further washed using 350 μL 1 × Washing Buffer thrice. Hundred microliters of Detection Antibody solution was added to the ELISA strips and incubated for 1 h at room temperature. ELISA wells were washed as mentioned above. Hundred microliters of the Avidin–HRP solution was added to all wells and incubated for 30 min at room temperature. ELISA wells were washed as mentioned above. Hundred microliters of Development Solution was added to each well and incubated for 15 min at room temperature in the dark. Hundred microliters of Stop Solution was added to each well. The color changed from blue to yellow. The absorbance at 450 nm was measured within 30 min of stopping the reaction. The estimation of pg/mL of cytokines was performed using the values obtained from antigen standards and a graph was plotted for the cytokine expression with the effect of treatments.

### Gene Expression Analysis

In order to detect the fold change in gene expressions quantitative real-time polymerase chain reaction (qRT-PCR, iQ-5, Bio-Rad, Australia) was used. RNA was isolated from the tissues using TRizol (Invitrogen, Australia) and isolated RNA had been immediately subjected to cDNA synthesis. The cDNA thus synthesized was stored at -80°C and was further used for a qRT-PCR chain reaction.

### Western Blotting

The tissues were homogenized and lysates were obtained by incubating homogenized tissue in radioimmunoassay precipitation (RIPA) buffer for 20 min at 4°C, followed by centrifugation at 14,000 rpm for 15 min at 4°C. The Bio-Rad Bradford assay (Bio-Rad Laboratories, Inc., Berkeley, CA, USA) was used to determine the protein concentration of the cellular extracts. Thirty milligrams of protein extracts were electrophoresed on 4–12% Bio-Rad gel. Transfer of proteins from the SDS–polyacrylamide gel onto the polyvinylidene difluoride (PVDF) membrane was carried out using Bio-Rad (Australia) *trans*-blot turbo transfer system. Following the transfer, blocking of membrane with 2% skimmed milk was performed for 1 h and the membrane was washed thrice with tris–buffer saline with tween 20 (TBST) and thrice with TBS. After the washing steps, the membrane was incubated with primary antibodies-mouse monoclonal anti-clathrin antibody (BD biosciences, 1:1000 dilution), rabbit anti-claudin antibody (1:1000 dilution, Santa Cruz, Australia), D8-mouse monoclonal anti-survivin, (Santa Cruz: 1:1000 dilution), mouse monoclonal TGF-beta-1 antibody (Santa Cruz: 1:1000 dilution), mouse monoclonal alpha-SMA antibody (Santa Cruz: 1:1000 dilution), mouse monoclonal Fibronectin-1 antibody (Santa Cruz: 1:1000 dilution), mouse monoclonal collagen type IV antibody (Santa Cruz: 1:1000 dilution), rabbit anti-GAPDH (Santa Cruz, 1:1000), overnight at 4°C. The washing steps were repeated and then incubated with the corresponding horseradish peroxidase (HRP) conjugated secondary antibodies (R&D biosciences rabbit anti-mouse HRP, goat anti-rabbit HRP: 1:1000 dilution) for 1 h at 37°C. The washing steps were repeated again and the membrane was developed using HRP substrates (GE healthcare, Australia). The membrane was visualized using Bio-Rad-Australia, Chemi-doc with XRS camera.

### Mean Opacity Measurements and Mean Permeability Measurements

The mean *in vivo* scores was determined using the mean opacity measurements. The rat eyes were excised carefully and carried to the laboratory in HBSS supplemented with penicillin/streptomycin. The measurement of mean opacity was carried out using an opacitometer which measures the transmission of light through the center of each mounted cornea. The mean permeability score was determined by adding a fluorescent (rhodamine solution) on the anterior side of the holder and the corneas were incubated in a horizontal position for 90 min at 32°C. The medium in the posterior chamber was removed and added to 96 well plates and the OD at 490 nm was read. The readings obtained at OD490 were used to determine the permeability of each treatment with respect to the change in the untreated corneas.

### Blood Analysis

The analysis of blood samples was performed directly using the ABX Micros ES 60 (Horiba Medicals). The parameters analyzed included the number of red blood cells (RBC’s), white blood cells (WBC’s), platelets and hemoglobin content. Briefly, 20 μL of the blood sample was acquired in the instrument and the readings were recorded.

### Pharmacokinetics

Pharmacokinetic analysis was done using the HPLC System for the levels of TSA in eye tissue and plasma samples was measured using PK methods. PK estimated using standard measurements for maximum concentration (*C*_max_), time to reach *C*_max_ (*T*_max_), area under the concentration–time curve (AUC), elimination rate constant (*K*), elimination half-life (*t*_1/2_), apparent clearance (CL/*F*), apparent volume of distribution (*V*_d_/*F*).

### Synthesis and Characterization of Alpha-SMA Targeted Ultra-small Chitosan Nanoparticles (USC NPs)

The synthesis of USC NPs was carried out using a previously published protocol (Sunkireddy et al., 2016). Briefly, 5.0 mL of aqueous STPP (2.0 mg/mL) was added drop-wise in 10.0 mL of low molecular weight chitosan (0.225% w/v in 1% v/v acetic acid) kept in a 25.0 mL beaker at room temperature while stirring at 600 rpm. The reactants were allowed to react under similar stirring conditions for 1 h after addition of STPP. To synthesize protein encapsulated ultra-small CS NPs, 5.0 mL of aqueous STPP (2.0 mg/mL) containing SurR9-C84A (4 mg/mL) was added drop-wise in 10.0 mL of chitosan kept on 25.0 mL beaker at room temperature. The components were stirred for 1 h after dropping STPP and SurR9-C84A to achieve the successful encapsulation of SurR9-C84A. These nanoparticles are not separable by centrifuging hence, in order to remove the unreacted STPP and unencapsulated proteins they were dialyzed using a 100 kDa dialysis tubing. The suspension was then added to 30 mg of EDC and 15 mg of NHS and stirred for 30 min (Roy et al., 2016). The alpha-SMA antibody suspension (100 μg/mL in 3 mL of PBS) was added dropwise at room temperature (25 ± 5°C) under sustained magnetic stirring for 12 h. The solution was dialyzed using a 100 kDa dialysis tubing to remove unreacted antibody and EDC or NHS.

Scanning electron microscopy (SEM, Supra 55vp Zeiss) was used to observe the surface morphology, size, and shape of the nanoparticles. To measure the thermal stability and water holding capacity of the nanoparticles differential scanning colorimeter (DSC, TA-Q series) was used. Fourier transform infra-red spectroscopy (FTIR) was used to study the chemical nature and complex structure of both void and alpha-SMA targeted USC NPs.

### *In Vitro* and *Ex Vivo* Internalization of Targeted USC NPs

The time-dependent *in vitro* NP internalization of the ultra-small CS NPs in MIOM1 (Müller glial cells) was determined using confocal microscopy. Cells were grown in Falcon^TM^ six-well culture slides (BD Biosciences, San Jose, CA, USA) at a density of 2 × 10^4^ cells/well and incubated with rhodamine-labeled ultra-small CS NPs at time points of 30 min. After the treatment, the media were discarded and cells were washed using PBS in order to remove the unbound NPs. The cells were fixed using 4% PF for 20 min. DAPI (4′,6-diamidino-2-phenylindole) was added to the cells and a cover slip was placed on the slide. The slide was viewed using the confocal microscope (Leica TCS SP5).

Eyes excised from humanly euthanized rats were transported to the laboratory in HBSS. Sterile PBS was used to wash the eyes and then eyes were incubated with 5 mg/ml of cyanine 5.5 labeled ultra-small CS NPs for 30 min. Following the treatment, eyes were carefully washed using PBS and imaged using Caliper Life Sciences *in vivo* imaging system using the Living Image 4.3.1 software (Sunkireddy et al., 2016).

### Statistical Analysis

Statistical analysis was performed by unpaired Student’s *t*-test using online Graphpad software on the triplicate data generated from individual or triplicate experiments. (^∗^) *P* < 0.05, (^∗∗^) *P* < 0.01 and (^∗∗∗^) *P* < 0.001.

## Results

### Regenerative and Protective Effects of TSA and SurR9-C84A

*Ex vivo analysis* was performed to determine the effective dosage of TSA and SurR9-C84A required to inhibit the expression of survivin, TGF-beta-1, alpha-SMA and collagen IV in alkali bunt cornea. It was observed that 0.02 mg/kg of TSA and 1.2 mg/kg of SurR9-C84A were most effective in inhibiting expression of survivin, alpha-SMA, TGF-beta-1 and collagen IV (**Figure 1A**). The rat alkali burn induced by NaOH led to extensive damage to the ocular surface and the anterior segment which induced ocular disfigurement and could also have caused a visual impairment (**Figure 1B**). The rat eyes were excised and examined for the pathological symptoms and according to the severity of damage a grading was performed (**Table 1**). The mean opacity, permeability, and irritation were measured in the rat eyes and the scores revealed that induction of alkali burn using NaOH led to a moderate irritation in rat eye whereas use of TSA and SurR9-C84A effectively led to a decrease in opacity, permeability and *in vitro* scores.

**Table 1:** Measure of opacity, permeability, and irritation.

Treatment	Mean opacity measurement	Mean permeability measurement	Mean *in vitro* score	Predicted irritation potential
Control eye	0.0	0.0	0.0	No irritation
Alkali burn (NaOH)	2.5 ± 0.5^∗∗^	0.80 ± 0.5^∗∗^	10.2 ± 0.5^∗∗∗^	Moderate irritation
NaOH–TSA	0.5 ± 0.2 ^∗^	0.15 ± 0.5^∗^	0.20 ± 0.5^∗^	No irritation
NaOH–SuR9-C84A	0.0^∗^	0.0^∗^	0.0^∗^	No irritation
NaOH–SuR9-C84A+TSA	0.0^∗^	0.0^∗^	0.0^∗^	No irritation

*Total number of animal (*n*) = 4. ^∗^*p* < 0.05, ^∗∗^*p* < 0.01 and ^∗∗∗^*p* < 0.001.*

The H&E sections (**Figure 1B**) obtained from the corneal segments clearly revealed that the NaOH insult led to damage of the conjunctival layer and the corneal matrix creating large vacuoles. Treatments with TSA and SurR9-C84A led to the recovery of the conjunctival layer, however, the corneal matrix was unable to heal completely. However, the combination of TSA and SurR9-C84A led to a substantial recovery as both the conjunctival layer as well as the corneal matrix showed improved physiology. Further analysis was carried out using immunofluorescence where the conjunctival integrity and corneal integrity was found to be severely damaged as the clathrin and claudin expressions (**Figure 1C**) respectively, were substantially lowered post-NaOH insult. SurR9-C84A was found to be more effective than TSA alone in reviving both the clathrin and claudin expressions in the conjunctival and corneal layer but the combination of both TSA and SurR9-C84A was found to most effective as the expressions of both these proteins were nearly same as that of control cornea. In order to confirm these findings western blotting was performed using the rat corneal lysates (**Figure 1D**). The results revealed that there was a substantial increase (1.947-fold) in clathrin expression in rat eyes treated with TSA+SurR9-C84A post-NaOH insult when compared to the alkali burnt eye. A similar trend was observed in the claudin expression where alkali insult led to a decrease in the claudin expression and there was a substantial increase (1.33-fold) in claudin expression in rat eyes treated with TSA+SurR9-C84A post-NaOH insult when compared to the alkali burnt eye (**Figure 1E**).

### Gene Expression Analysis of Key Wound Healing Proteins and Inflammatory Cytokines

**FIGURE 2:**
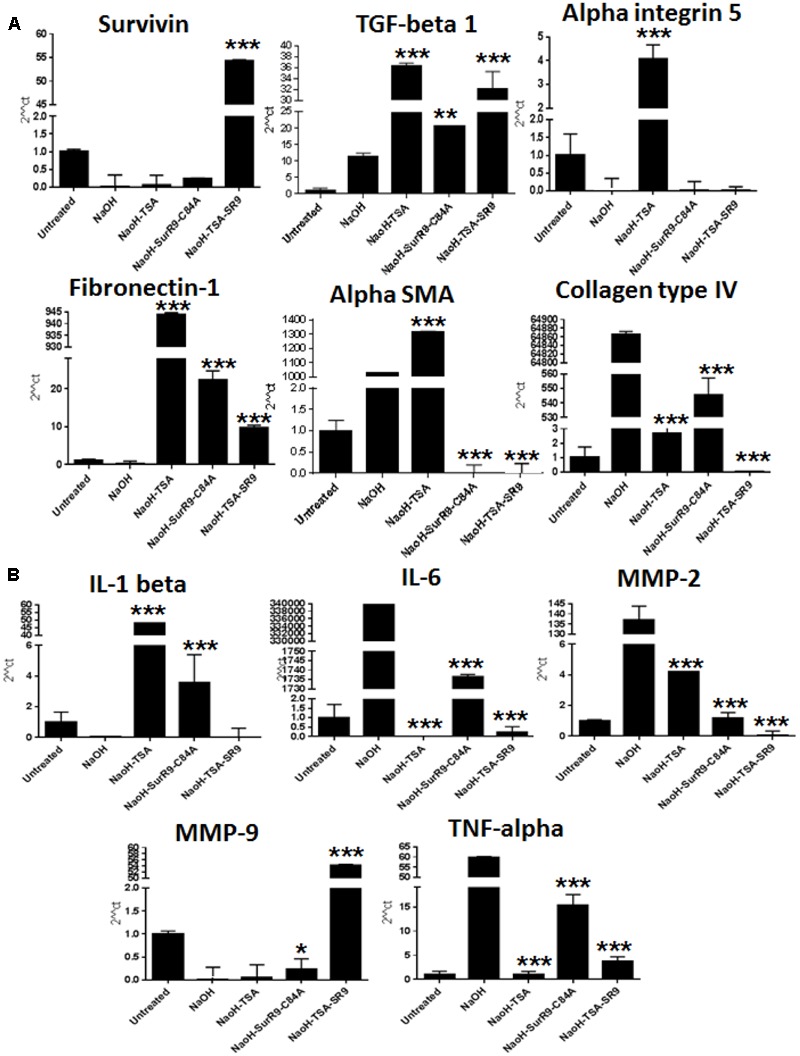
**Quantitative real-time PCR for expression of wound healing, tissue integrity and inflammatory markers: (A,B)** The qRT-PCR analysis was carried out to determine the gene expressions of wound healing markers survivin, TGF-beta-1, tissue integrity markers alpha integrin 5, Fibronectin-1, alpha-SMA, collagen type IV and inflammatory markers IL-1 beta, IL-6, MMP-2, MMP-9 and TNF-alpha. (SR9=SurR9-C84A). The experiments were conducted in triplicates. ^∗^*p* < 0.05, ^∗∗^*p* < 0.01, and ^∗∗∗^*p* < 0.001.

Gene expression analysis was carried out to determine the expressions of key regulators of cell survival, morphology wound healing, and inflammation. The qRT-PCR analysis revealed that survivin expression was significantly increased (*p* < 0.001) in rat eyes treated with SurR9-C84A and TSA+SurR9-C84A post-alkali burn. TGF-beta-1 expression was found to increase post-NaOH insult and was found to further increase with TSA (*P* < 0.001) and SurR9-C84A treatments (*p* < 0.01). The alpha-integrin 5 expression was lowered post-NaOH insult whereas, treatments with TSA led to a significant increase (*p* < 0.001) in both alpha-integrin 5 and alpha-SMA (*p* < 0.001) expressions. SurR9-C84A and TSA+SurR9C84A, on the other hand, led to a significant decrease (*p* < 0.001) in alpha-SMA expression. Fibronectin-1 expression was also found to be significantly enhanced by TSA (*p* < 0.001), SurR9-C84A (*p* < 0.001) and TSA+SurR9-C84A (*p* < 0.001). Insult with NaOH led to an increase in Collagen type IV expression which was found to be efficiently downregulated by TSA (*p* < 0.001), SurR9-C84A (*p* < 0.001) and TSA+SurR9-C84A (*p* < 0.001) (**Figure 2A**).

IL-1 beta expression was found to be decreased with NaOH insult and both TSA (*p* < 0.001) and SurR9-C84A (*p* < 0.001) led to an increase in its expression. However, TSA+SurR9-C84A did not lead to any increase in IL-1 beta. IL-6 and TNF-alpha expressions were found to be increased post-insult with NaOH and were found to be significantly lowered with TSA (*p* < 0.001) alone followed by TSA+SurR9-C84A treatments (*p* < 0.001). MMP-2 expression was also found to increase with NaOH and treatments and TSA+SurR9-C84A was the most effective in downregulating (*p* < 0.001) MMP-2 followed by SurR9-C84A and TSA. MMP-9 expression was lowered post-NaOH treatments whereas, treatments with TSA+SurR9-C84A led to a significant increase (*p* < 0.001) in MMP-9 expression (**Figure 2B**).

### Ocular and Systemic Pharmacokinetics of TSA

Trichostatin-A concentration was determined after topical application of TSA and TSA-SurR9-C84A in rat eyes and plasma (**Tables 2, 3**). The maximum concentration (*C*_max_) of TSA in different ocular tissue and plasma were measured. It was also found that the elimination half-life of TSA in cornea, conjunctiva, and aqueous humor was relatively longer than that in tear fluid and plasma.

**Table 2:** Concentration of TSA in tissues and plasma after topical application in eye.

Time (min)	Plasma (ng/mL, mean ±*SD*)	Aqueous humor (ng/mL, mean ±*SD*)	Cornea (μg/g, mean ±*SD*)	Conjunctiva (μg/g, mean ±*SD*)	Tear fluid (μg/g, mean ±*SD*)
2	103.61 ± 28.37	119.43 ± 37.92	216.83 ± 78.05	267.88 ± 75.51	333.42 ± 69.72
10	263.87 ± 80.96	112.94 ± 15.10	291.02 ± 109.25	261.29 ± 66.47	208.93 ± 30.82
30	109.16 ± 33.00	62.80.24 ± 7.29	762.86 ± 385.68	178.23 ± 35.22	154.98 ± 73.25

*Peaks were referenced with commercial standard from Assay Matrix Pty Ltd.

**Table 3:** Pharmacokinetics of TSA in rat eye tissues and plasma after topical application.

Pharmacokinetic parameters	Plasma^a^	Aqueous humor^b^	Cornea	Conjunctiva	Tear fluid
*C*_max_ (μg/g)	160.3 ± 61.0	11,046 ± 2734	44.67 ± 12.91	26.26 ± 7.19	2,987 ± 1814
*T*_max_ (h)	0.167	0.333	0.0833	0.0833	0.0833
*t*_1/2_ (h)	1.5	1.5	3.5	4.2	2.3
CL/*F* (μg/h)	12.6	0.5	7.5	11.5	1.0
*V*_d_/*F* (μg)	34.5	0.6	35.5	45.5	2.0
AUC_0-8_ (μg h/g)	65.5	7568	45.5	53.4	678.5
AUC_0-∞_ (μg h/g)	68.4	7689	55.5	51.70	768.5

*Apparent clearance = CL*/F* and apparent volume of distribution = *V*_*d*_*/F*. ^*a,b*^Unit for plasma/aqueous humor = ng/mL, *C*_*max*_ = L/h, L/ng h/mL and AUC = CL/*F, V*_*d*_/F.*

### Effect of Combination of TSA and SurR9-C84A on Corneal Membrane Integrity Post-alkali Burn

**FIGURE 3:**
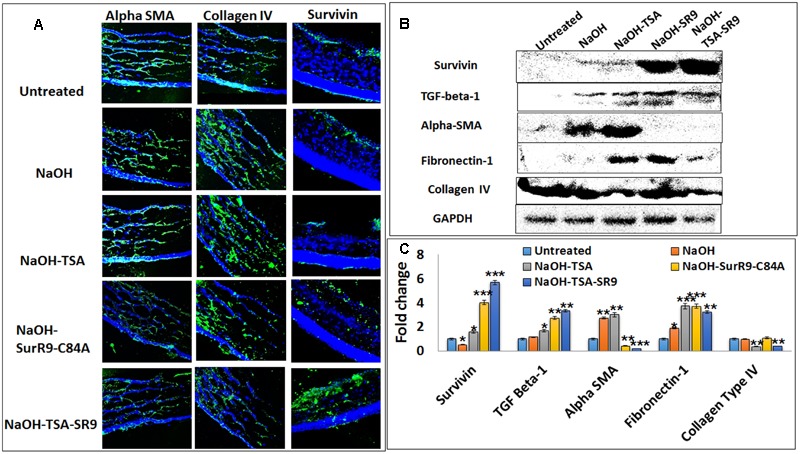
**Analysis of protein expression for key tissue integrity markers: (A)** Immunofluorescence staining was performed for expression of alpha-SMA, collagen type IV and survivin in rat corneal tissues. **(B)** Western blotting analysis was performed to confirm the protein expressions of survivin, TGF-beta-1, alpha-SMA, Fibronectin-1 and collagen type IV in rat corneal lysates. Each blot was repeated thrice and a representative blot has been presented. **(C)** Band density analysis was carried out for the western blots using the image J software. (SR9=SurR9-C84A). Five images of each treatment were taken for immunofluorescence studies and a representative image has been presented. ^∗^*p* < 0.05, ^∗∗^*p* < 0.01, and ^∗∗∗^*p* < 0.001.

Confocal microscopy was carried out in order to determine the protein expressions of key proteins regulating cell morphology (**Figure 3A**). Survivin expression was found to be slightly increased post-NaOH insult and treatments with SurR9-C84A and TSA+SurR9-C84A led to an increase in survivin expression. It was observed that alpha-SMA expression was substantially enhanced post-NaOH insult and TSA treatments failed to downregulate it effectively. SurR9-C84A and TSA+SurR9-C84A led to substantial decrease in alpha-SMA expression. The expression of collagen type IV was found to be downregulated by NaOH whereas treatments with TSA were found to be the most effective in increasing the collagen type IV expression followed by TSA and TSA+SurR9-C84A. In order to confirm these findings western blotting was performed to determine the expression of key proteins (**Figure 3B**). The results revealed that survivin expression was substantially increased post-SurR9-C84A (7.7-fold) and TSA+SurR9-C84A (13.1-fold) treatments. TGF-beta-1 expression was found to be substantially increased post-TSA+SurR9-C84A (threefold) followed by SurR9-C84A (2.4-fold) and TSA (1.45-fold) treatments. Alpha-SMA expression was found to be substantially increased post-NaOH insult (2.7-fold). Treatments with SurR9-C84A (6.75-fold) and TSA+SurR9-C84A (15-fold) led to an effective downregulation of alpha-SMA. Fibronectin-1 expression was found to be significantly enhanced by TSA (1.97-fold), SurR9-C84A (1.97-fold) followed by TSA+SurR9-C84A treatments (1.71-fold). In the case of collagen type IV, a slight decrease was observed with NaOH insult (1.1-fold) and treatments with TSA (2.8-fold) and TSA+SurR9-C84A (2.42-fold) led to an effective downregulation of collagen type IV (**Figure 3C**).

### Analysis of Key Inflammatory and Anti-inflammatory Cytokines in Alkali Burn

**FIGURE 4:**
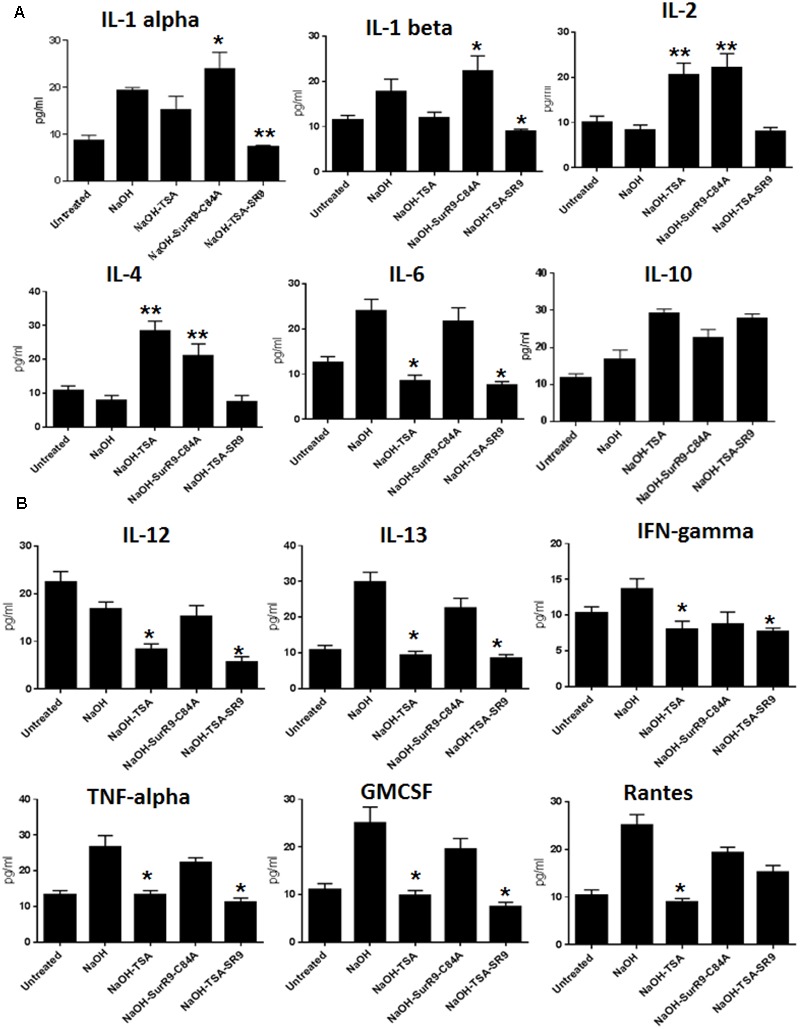
**ELISA for expression of pro-inflammatory and anti-inflammatory cytokines: (A,B)** The ELISA array was performed to determine the expression of pro-inflammatory cytokines IL-1 alpha, IL-1 beta, IL-2, IL4, IL-6, IL-12, IFN-gamma, TNF-alpha and Rantes and anti-inflammatory cytokines such as IL-10, IL-13, GMCSF. (SR9=SurR9-C84A). The experiments were conducted in triplicates. ^∗^*p* < 0.05 and ^∗∗^*p* < 0.01.

Multianalyte ELISA test was conducted to determine the expression of inflammatory cytokines in the corneal lysate. The results revealed that IL-2 expression was significantly increased (*p* < 0.01) with treatments of TSA and SurR9-C84A. In the case of IL-4 NaOH treatments led to a decrease in expression. The expression of IL-4 was found to increase with treatments of TSA (*p* < 0.01) and SurR9-C84A (*p* < 0.01). The IL-1 alpha expression was found to significantly increase with SurR9-C84A (*p* < 0.05) treatments and was significantly reduced by TSA+SurR9-C84A (*p* < 0.05) treatments. The IL-1 beta expression was also found to increase post-NaOH insult. Treatments with SurR9-C84A led to a further increase (*p* < 0.05) in IL-1 beta whereas, treatments with TSA and TSA+SurR9-C84A led to a significant (*p* < 0.05) downregulation. A similar pattern in expressions of IL-6, IL-13, TNF-alpha and GMCSF was observed. Their expression was enhanced with NaOH insult and downregulated effectively by both TSA and TSA+SurR9-C84A (*p* < 0.05) treatments. In the case of IL-10, NaOH led to a significant increase (*p* < 0.01) and treatments of TSA+SurR9C84A led to an effective decrease (*p* < 0.05) in its expression (**Figure 4A**). The IL-12 expression was decreased by NaOH insult and treatments with TSA and TSA+SurR9-C84A led to a further downregulation (*p* < 0.05) in the expression of IL12. IFN-gamma was found to be upregulated with NaOH insult and treatments with TSA and TSA+SurR9-C84A led to a significant downregulation (*p* < 0.05) in the expression of IFN-gamma. It was also observed that Rantes was upregulated by NaOH insult and significantly downregulated by TSA (*p* < 0.05) treatments (**Figure 4B**).

### Histopathology and Blood Analysis for Signs of Toxicity

**FIGURE 5:**
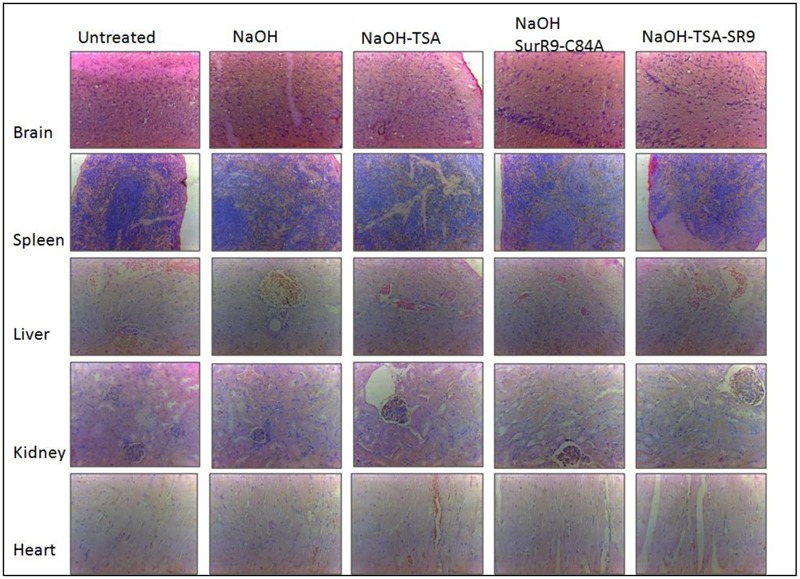
**Histology analysis for signs of toxicity: The vital organs from treated and control rats such as brain, spleen, liver, kidney and heart were analyzed using H&E staining for signs of toxicity post-insult with NaOH and treatments with TSA, SurR9-C84A and TSA+SurR9-C84A (SR9).** The H&E staining was performed on five slides obtained from each animal and a representative image has been presented.

The histopathology of key organs such as brain, spleen, liver, kidney, and heart was conducted in order to observe any signs of cytotoxicity or adversity due to any of the treatments (**Figure 5**). No substantial change was observed in the tissue sections from all organs of the treated rats. The blood analysis was carried out to determine the effect of alkali burn and the treatments on various parameters of blood (**Table 4**). It was observed that in the untreated rats the hemoglobin (HGB) count was higher than normal, this count was decreased by NaOH insult whereas treatments with TSA+SurR9-C84A led to an increase in HGB. The hematocrit (HCT) count was found to be lower than normal range in the case of NaOH treated rats. Treatments with TSA, SurR9-C84A, and TSA+SurR9-C84A effectively increased the HCT count to normal range. The mean corpuscular HGB count was found to be higher than normal in NaOH treated rats which were effectively reduced to normal range upon treatments with TSA, SurR9-C84A, and TSA+SurR9-C84A. The platelet count in untreated rats was found to be low in untreated rats and was increased by treatments with NaOH, SurR9-C84A, and TSA+SurR9-C84A.

**Table 4:** Blood analysis.

Parameters	Untreated	NaOH	NaOH–TSA	NaOH–SurR9-C84A	NaOH–TSA–SurR9-C84A
WBC	8.15 ± 2.02	3.4 ± 1.19	5.48 ± 2.06	1.3 ± 3.18	8.8 ± 2.43
RBC	9.10 ± 0.41	6.53 ± 2.89	8.15 ± 0.61	8.35 ± 0.99	9.51 ± 0.82
HGB	18.66 ± 1.01	14.61 ± 5.35	17.61 ± 1.41	17.1 ± 1.11	18.75 ± 1.69
HCT	50.71 ± 2.36	40.71 ± 1.76	46.2 ± 4.14	46.85 ± 4.14	52.98 ± 4.07
MCV	55.66 ± 0.51	62.5 ± 8.01	56.66 ± 1.86	55.66 ± 1.86	47.5 ± 2.08
MCH	20.5 ± 0.55	23.45 ± 4.36	21.63 ± 1.01	20.36 ± 1.42	19.73 ± 0.70
MCHC	36.81 ± 1.39	37.51 ± 4.79	38.15 ± 1.63	36.61 ± 1.57	35.58 ± .04
PLT	334.16 ± 116	487 ± 166	256.33 ± 117	547.16 ± 130	409.66 ± 135

*WBC, white blood cells; RBC, red blood cells; HGB, hemoglobin; HCT, haematocrit; MCV, mean corpuscular volume; MCH, mean corpuscular hemoglobin; MCHC, mean corpuscular hemoglobin concentration; PLT, platelets.*

### Characterization of Alpha-SMA Targeted Ultra-Small Chitosan Nanoparticles (USC NPs)

**FIGURE 6:**
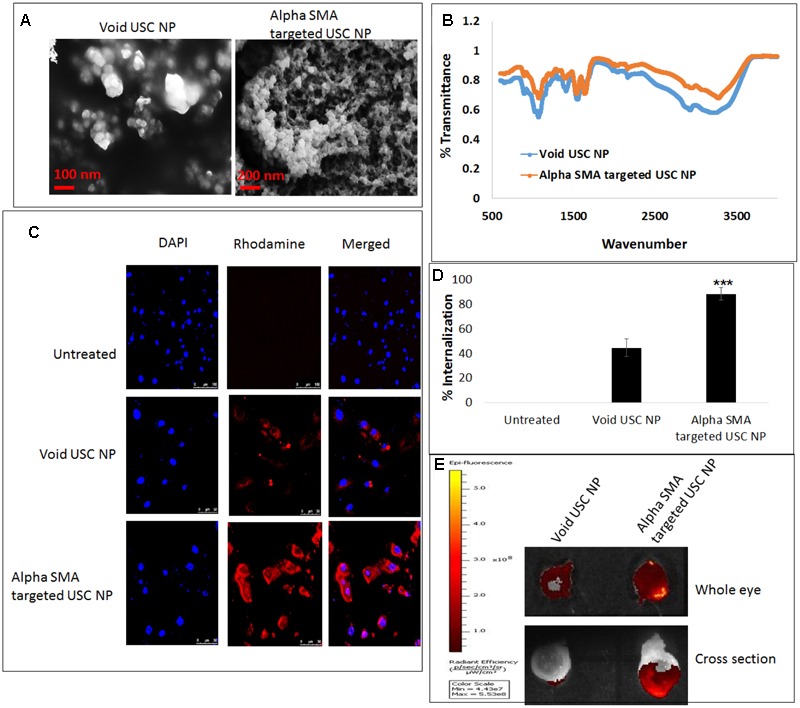
***In vitro* and *ex vivo* internalization efficacy of alpha-SMA targeted USC NPs: (A)** SEM images confirmed uniform shape and size (50–60 nm) of both void and alpha-SMA targeted USC NPs. **(B)** FTIR analysis confirmed successful conjugation of anti-body represented by the sharp primary amine peak at 1640 cm^-1^ in spectra of alpha-SMA targeted USC NPs. **(C)** Confocal microscopy revealed that alpha-SMA targeted USC NPs showed substantially higher internalization in MIOM-1 cells when compared to void USC NPs. **(D)** Graphical representation of confocal images showing internalization efficacy of alpha-SMA targeted USC NPs over void USC NPs. **(E)** *Ex vivo* internalization analysis also confirmed that alpha-SMA targeted USC NPs showed higher internalization efficacy than void USC NPs. The experiments were conducted in triplicates. Five images of each treatment were taken for immunofluorescence studies and a representative image has been presented. ^∗∗∗^*p* < 0.001.

The USC NPs were synthesized and characterized using SEM (**Figure 6A**) and FTIR. The infra-red spectrum of void USC NP was compared to that of alpha-SMA targeted USC NPs using FTIR. For void USC NPs, the characteristic NH_2_ and OH group stretching peak was observed at 3425 cm^-1^. The peaks were wider in the case of alpha-SMA targeted USC NPs (3432 cm^-1^) suggesting an increase in the number of OH bonds. The NH_2_ stretching peak for void USC NPs was observed at 1640 and 1559 cm^-1^, and for alpha-SMA targeted USC NPsat 1403 cm^-1^, the peaks for targeted USC NPs were again broad suggesting an interaction between NH_3_^+^ of chitosan and STPP. The CH deformation vibration peak for void USC NPs was at 1412 cm^-1^ whereas for targeted USC NPs nanoparticles were at 1421 cm^-1^ and shorter than that of void NPs. The P=O group peak was observed at 1095 cm^-1^ for void NPs, and at 1098 cm^-1^ for alpha-SMA targeted USC NPs (**Figure 6B**).

### *In Vitro* and *Ex Vivo* Internalization Efficacy of Alpha-SMA Targeted USC NPs

Confocal microscopy was conducted in order to test the internalization of Void USC NPs and alpha-SMA targeted USC NPs in MIOM1 cells (**Figure 6C**). It was observed that post 30 min of incubation the void NPs showed successful uptake in MIOM1 cells, however, a higher and more efficient internalization was observed in the case of alpha-SMA targeted USC NPs (**Figure 6D**).

The internalization efficacy was also carried out in an *ex vivo* rat eye model. The nanoparticles were incubated with whole rat eyes for 30 min and post-washing, the eyes were examined using IVIS. It was evident that the rat eyes treated with alpha-SMA targeted USC NPs showed higher fluorescence intensity than the void USC NPs. The eyes were then frozen overnight and cross-sectionally segmented. The cross-sectional sections were also examined using IVIS and it was found that a high intensity of fluorescence intensity proportional to alpha-SMA targeted USC NPs was observed (**Figure 6E**).

## Discussion

Several studies have focused on control and prevention of alkali burn as it’s a very common and has severe effects (Pattamatta et al., 2013; Ambrosone et al., 2014; Okada et al., 2014; Yang et al., 2015). Alkali burns can destroy not only the corneal epithelium but also the corneal stroma, by creating large vacuolated structures (Zhang et al., 2005). In the present study, large vacuoles were found in the corneal stroma in eyes post-NaOH insult. In our previous study, we found that combination treatment of dominant negative survivin mutant (SurR9-C84A) and histone deacetylase inhibitor trichostatin-A (TSA) is effective for maintaining the corneal tissue’s integrity in rabbit alkaline burn model (Roy et al., 2016). Moreover, including additional biomarkers of the disease pathway in the proposed rat model, will give a better understanding of haze/scarring mechanisms and will provide more information on the effectiveness of the treatment for future work in human trials. Treatments with TSA, SurR9-C84A, and TSA+SurR9-C84A led to healing of both corneal epithelium and corneal stroma as seen by the images from the eye and the H&E sections. It has been reported earlier that TSA led to the recovery of rat corneal cells within a span of just 2 min of topical administration after induction of alkali burn (Sharma et al., 2009). Clathrin protein is generally found in epithelial cells of conjunctiva and is responsible for regulation of intracellular segregation and transportation of matter into the eye (Qaddoumi et al., 2003). Thus, during ocular drug delivery change in expression of conjunctival clathrin has been related to conjunctival inflammation and damage (Gukasyan et al., 2008). Claudin transmembrane protein that acts as a barrier molecule by regulating the cell–cell interaction and tight junctions along with tight junction protein ZO-1, to maintain the membrane structure of corneal epithelial cells (Peltonen et al., 2007). Claudin is responsible for sealing the adjacent cells and tightening of the paracellular spaces to prevent the passage of the solutes through the corneal membranes (Krause et al., 2008; Contreras-Ruiz et al., 2012). Therefore, disruption of claudin is a sign of corneal toxicity and damage. Both clathrin and claudin expressions were found to be substantially lowered by NaOH treatments, whereas further treatments with TSA+SurR9-C84A (found to be the most effective) led to conjunctival and corneal healing by reviving the expressions of both clathrin and claudin.

Alkali burn has been reported to downregulate expressions of alpha integrins, fibronectins, and alpha-SMA (Fish and Davidson, 2010). Fibronectin has a favorable effect on promoting epithelialization (Singh et al., 2013). Treatments of TSA post-NaOH was found to be the most effective in increasing fibronectin expression followed by SurR9-C84A and TSA+SurR9-C84A. During wound healing, the keratocytes that migrate to injured stroma transform into myofibroblasts. These myofibroblasts express high levels of alpha-SMA (Ishizaki et al., 1993). Although treatments of TSA led to an increase in expressions of alpha-SMA, treatments with SurR9-C84A and TSA+SurR9-C84A did not increase the alpha-SMA levels to very high. Expression of collagen type IV is generally determined to investigate the changes in the corneal epithelial basement membrane following an alkali burn (Saika et al., 1995). It was observed that collagen type IV expression was significantly increased post-NaOH insult but treatments with TSA and TSA+SurR9-C84A lowered it effectively. Increased collagen type IV has been often related with increased corneal haze (Kaji et al., 2001).

Alkali burn is known to generate a severe inflammatory response and attract the inflammatory cells toward the site of injury (Kenyon, 1985). Continued infiltration of the inflammatory cells can suspend the corneal re-epithelialization, and lead to ulceration and permanent visual loss (Kenyon, 1979, 1982). The exact profile of the various cytokines and their relative expressions during damage and repair processes in the alkali-burned cornea is yet to be elucidated. Research done over the past several years has made it clear that corneal epithelial cells can express and produce IL-1beta (Wilson et al., 1992); corneal epithelial, stromal and endothelial cells can produce IL-6 (Sakamoto et al., 1991); and corneal epithelial and stromal cells can express and produce IL-8 (Cubitt et al., 1993). The wound healing process, on the other hand, is regulated by several signals initiated from these cytokines.

IL-1beta, IL-6, and TNF-alpha are commonly known to be expressed in alkali-burned corneas, and IL-6, in particular, has been reported to be strongly induced soon after alkali burn injury in mice (Sotozono et al., 1997). It has also been reported that IL-1 alpha was predominantly present in regenerated corneal epithelial cells, especially in basal cells (Sotozono et al., 1997). Wilson et al. (1996) have suggested that IL-1 alpha and IL-1beta are inflammatory modulators released from the injured epithelium that serves as injury transducers, leading to keratocyte apoptosis. Because IL-1alpha and IL-1beta use the same receptors in a variety of cells, it is assumed that they have similar biological activities (Dinarello, 1991).

IL-6 is generally not expressed in unburned corneal epithelium (Sotozono et al., 1997). However, in alkali burnt corneas increased expressions of IL-6 have been reported (Sotozono et al., 1997). A dramatic increase in expressions of IL-1 alpha and IL-6 has been reported during the early stages of alkali burn (Li et al., 2001). Inflammatory cytokines such as IL-1, IL-6 and IL-8 lead to infiltration of polymorphonuclear leukocytes (PMNs) that produce more inflammatory cytokines. The PMNs also produce superoxide, prostaglandins and release lysosomal enzymes. IL-1 has also been reported to induce the expression of MMP family of enzymes in alkali burnt corneal stromal cells (Li et al., 2001), that further leads to corneal and stromal damage. Thus, it can be assumed that IL-1 is the initiator of the inflammatory response or the trigger to the cascade of pathways associated with corneal damage post-alkali burn. MMPs such as MMP-2 and MMP-9 are zinc-dependent extracellular endoproteinases that disrupt cell-to-cell and cell-to-extracellular matrix connections and facilitate the migration of endothelial cells to areas stimulated by VEGF, ultimately leading to the formation of new vasculature and redness of the eye (Kvanta et al., 2000).

Histone deacetylase inhibitors effect the cell death by altering the acetylation of non-histone proteins therefore, it is reported that they may be anti-inflammatory (Adcock, 2007). A study has shown that TSA substantially downregulated both the mRNA and protein expressions of the proinflammatory cytokines, such as tumor necrosis factor TNF-alpha, IL-6, and IL-1beta, on the other hand, pretreatment with TSA induced an increase in the expression of the immunosuppressive cytokine IL-10 (Han and Lee, 2009). Dominant negative survivin protein (SurR9-C84A) on the other hand has shown promising anti-inflammatory and chondroprotective activity against cartilage degradation and arthritis (Samarasinghe, 2013). SurR9-C84A treatments have also shown improved neuronal health following amyloid-beta (Aβ) peptide fragment and *N*-methyl-D-aspartate (NMDA) induced toxicity to brain cells in addition to inflammatory insults induced in mono and co-cultures of brain cells in an Alzheimer’s disease (AD) model (Sriramoju et al., 2015). The BIR motif of mutant survivin (SurR9-C84A) has been reported to bind with microtubules and control their stability, instigate cell division, enhance proliferation and trigger the expression of the cell cycle to induce proliferation of neurons and to protect them from apoptosis (Baratchi et al., 2010). Moreover, treatments of SurR9-C84A have also been reported to protect the neuronal cells from oxidative damage and peroxide-induced oxidative stress (Baratchi et al., 2011b). It has also been reported that SurR9-C84A acts as a neuroprotectant against the cytotoxic effects of activated T-cell infiltrates, such as granzyme B (GrB) (Baratchi et al., 2011a). Therefore, there is enough substantial evidence on the anti-inflammatory, protective and proliferative nature of both TSA and SurR9-C84A.

**FIGURE 7:**
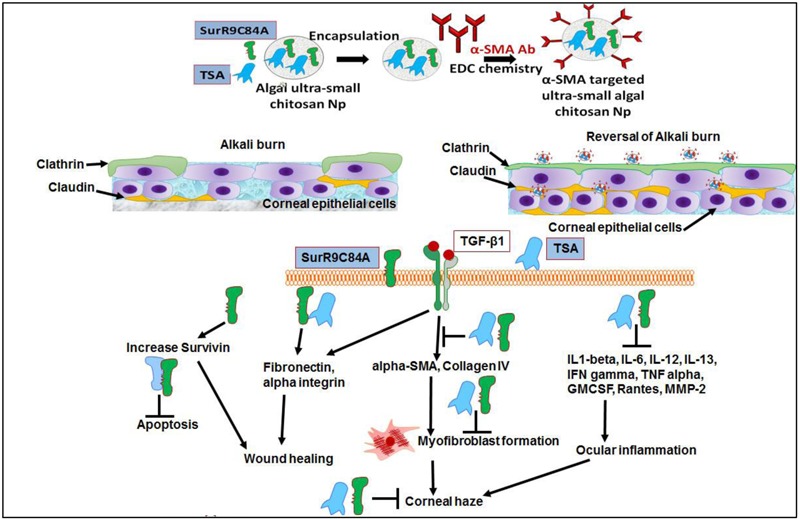
**Alpha-SMA targeted USC NPs for delivery of TSA and SurR9-C84A to the burn site and reverses corneal haze by inducing apoptosis in TGF-beta-1 induced transformed myofibroblast cells: The alpha-SMA targeted USC NPs have the potential for specifically delivering majority of the therapeutic to the burn site in a sustained release manner thus ensuring a longer activity period of the therapeutics.** The combinatorial treatment of SurR9-C84A and TSA can repair of the conjunctival and corneal integrity by reinstating expression of clathrin and claudin without inducing any non-specific cytotoxicity. TSA and SurR9-C84A treatment increased expression of survivin: preventing apoptosis, TGF-beta-1: initiating wound healing, fibronectin, and alpha integrin: reinstating the corneal integrity. Key markers of corneal haze progression (alpha-SMA and collagen type IV) were effectively downregulated therefore reducing the risk of myofibroblast formation.

The findings from the present study confirmed that combined treatment of SurR9-C84A and TSA lead to the repair of the conjunctival and corneal integrity by reinstating expression of clathrin and claudin without inducing any non-specific cytotoxicity. TSA and SurR9-C84A treatment increased expression of survivin-preventing apoptosis, TGF-beta-1-initiating wound healing, fibronectin, and alpha integrin–reinstating the corneal integrity. Key markers of corneal haze progression (alpha-SMA and collagen type IV) were effectively downregulated (**Figure 7**). TSA and SurR9-C84A treatment further led to the suppression of pro-inflammatory markers IL-1beta, IL-6, IL-12, IL-13, IFN-gamma, TNF-alpha, GMCSF, Rantes, and MMP-2. Thus this combination showed a promising healing and anti-inflammatory effect in a very short time post-alkali burn. Hence, this combination can prove to be a promising ophthalmic formulation for immediate response for wound healing post-alkali burnt cornea.

## Author Contributions

The authors would like to mention that KR performed the experiments and wrote the manuscript. RK and JK contributed to the concept and design of the study, data interpretation, data analysis and revision of the manuscript. CHAC and RS analyzed data and corrected the manuscript. PN revised the manuscript and analyzed HPLC data.

## Conflict of Interest Statement

The authors declare that the research was conducted in the absence of any commercial or financial relationships that could be construed as a potential conflict of interest.
