# Expression of Concern: Topical Ophthalmic Formulation of Trichostatin A and SurR9-C84A for Quick Recovery Post-alkali Burn of Corneal Haze

**DOI:** 10.3389/fphar.2017.00427

**Published:** 2017-06-28

**Authors:** Frontiers Editorial Office

With this notice, Frontiers states its awareness of serious allegations surrounding the article “Topical Ophthalmic Formulation of Trichostatin A and SurR9-C84A for Quick Recovery Post-alkali Burn of Corneal Haze” published on 5 May 2017. Our Chief Editors will direct an investigation in full accordance with our procedures. The situation will be updated as soon as the investigation is complete.

