# Corrigendum: Topical Ophthalmic Formulation of Trichostatin A and SurR9-C84A for Quick Recovery Post-alkali Burn of Corneal Haze

**DOI:** 10.3389/fphar.2018.01434

**Published:** 2018-12-18

**Authors:** Kislay Roy, Prasad Neerati, Chun Hei Antonio Cheung, Rupinder K. Kanwar, Rajat Sandhir, Jagat R. Kanwar

**Affiliations:** ^1^Nanomedicine-Laboratory of Immunology and Molecular Biomedical Research, Centre for Molecular and Medical Research, School of Medicine, Faculty of Health, Deakin University, Geelong, VIC, Australia; ^2^Drug Metabolism & Clinical Pharmacokinetics Division, Department of Pharmacology, University College of Pharmaceutical Sciences, Kakatiya University, Warangal, India; ^3^Department of Pharmacology and Institute of Basic Medical Sciences, College of Medicine, National Cheng Kung University, Tainan, Taiwan; ^4^Department of Biochemistry, Panjab University, Chandigarh, India

**Keywords:** survivin, haze, scarring, wound healing, cytokines, trichostatin-A

Immediately after publication of the original article, the authors found that the incorrect versions of Figure 1B and Table 2 were included due to human error. The correct version of Figure 1 and Table 2 are included in this Corrigendum.

**Figure 1:**
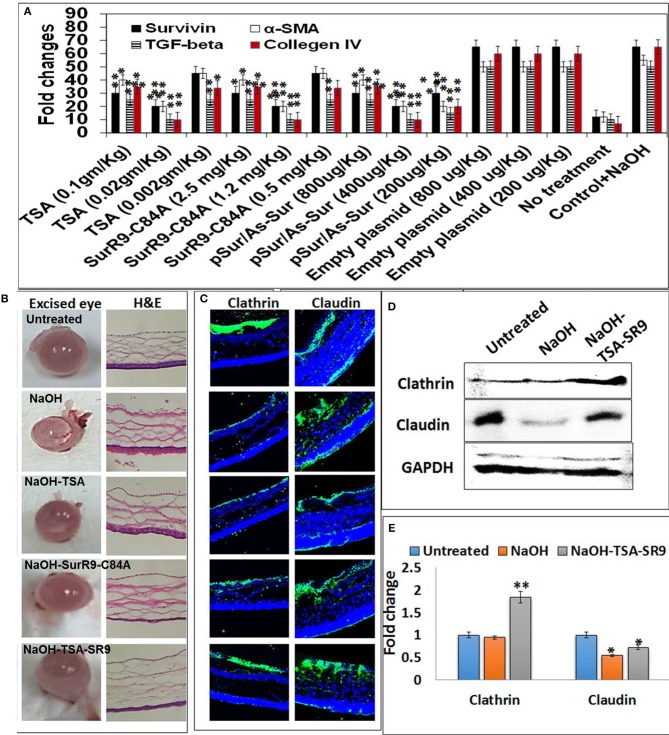
Change in corneal tissue integrity post-alkali burn: **(A)** Gene expression analysis to determine the effective dosage of TSA and SurR9-C84A using *ex vivo* rat eyes. **(B)** Rat eyes were exposed to 1 M NaOH for 1 min and then treated with TSA or SurR9-C84A or a combination of both [TSA+SurR9-C84A(SR9)] for 40 min. The excised eyes were used to determine the opacity and inflammatory scores and H&E staining was performed to determine the effect of alkali burn and treatments on corneal tissue integrity. **(C)** Immunofluorescence staining for expression of clathrin in conjunctival layer and claudin in corneal layer was performed to determine the effect of alkali burn and treatments on corneal tissue integrity. **(D)** Western blotting analysis confirmed that both decrease in expressions of clathrin and claudin in rat corneal tissue lysates. Each blot was repeated thrice and a representative blot has been presented. **(E)** Band density analysis of western blot was performed using image J software. The experiments were conducted in five animals per treatment group. The H&E staining was performed on five slides obtained from each animal and a representative image has been presented. Five images of each treatment were taken for immunofluorescence studies and a representative image has been presented. ^*^p < 0.05, ^**^p < 0.01, and ^***^p < 0.001.

**Table 2:** Concentration of TSA in tissues and plasma after topical application in eye^*^.

**Time (min)**	**Plasma** **(ng/mL, mean ±*SD*)**	**Aqueous humor** **(ng/mL, mean ±*SD*)**	**Cornea** **(μg/g, mean ±*SD*)**	**Conjunctiva** **(μg/g, mean ±*SD*)**	**Tear fluid** **(μg/g, mean ±*SD*)**
2	103.61 ± 28.37	119.43 ± 37.92	216.83 ± 78.05	267.88 ± 75.51	333.42 ± 69.72
10	263.87 ± 80.96	112.94 ± 15.10	291.02 ± 109.25	261.29 ± 66.47	208.93 ± 30.82
30	109.16 ± 33.00	62.80.24 ± 7.29	762.86 ± 385.68	178.23 ± 35.22	154.98 ± 73.25

* *Peaks were referenced with commercial standard from Assay Matrix Pty Ltd*.

Prasad Neerati was not included as an author in the published article. HPLC data in Table 2 was repeated and now Prasad Neerati is included in the list of authors in the article. The authors apologize for these errors and state that this does not change the scientific conclusions of the article in any way. The original article has been updated.

## Conflict of Interest Statement

The authors declare that the research was conducted in the absence of any commercial or financial relationships that could be construed as a potential conflict of interest.

